# Increased final adult height by gonadotropin-releasing hormone agonist in girls with idiopathic central precocious puberty

**DOI:** 10.1371/journal.pone.0201906

**Published:** 2018-08-22

**Authors:** Hae Sang Lee, Jong Seo Yoon, Kyu Jung Park, Jin Soon Hwang

**Affiliations:** Department of Pediatrics, Ajou University School of Medicine, Ajou University Hospital, Suwon, Korea; Florida International University, UNITED STATES

## Abstract

**Objective:**

Gonadotropin-releasing hormone agonists (GnRHa) are the treatment of choice for central precocious puberty (CPP) and have been widely used for several decades. We determined the effect of GnRHa treatment on the auxological outcomes of girls with idiopathic CPP.

**Methods:**

This study included 84 girls treated monthly with depot leuprolide acetate who had reached adult height. We compared their final adult height (FAH) with their initial predicted adult height (PAH). We performed a multivariate analysis of the factors associated with FAH on all girls diagnosed with CPP.

**Results:**

We performed the final evaluations at a mean age of 14.1 ± 0.8 years after a mean treatment duration of 2.98 ± 0.73 years (ranging from 1.5–4.8 years). Menarche had occurred at 12.6 ± 0.6 years of age, which was 16.5 ± 6.1 months after discontinuation of GnRHa therapy. Mean FAH was 160.1 ± 5.0 cm, which was significantly higher than the initial PAH (156.1 ± 5.7 cm; *P* < 0.001). To investigate whether growth outcomes were influenced by the age at initial treatment, we divided all patients into two groups, those treated between 6 and 8 years (n = 23) and those treated after 8 years (n = 61); no significant differences were observed in FAH between the two groups. FAH was significantly and positively correlated with the height standard deviation score (SDS) at the end of treatment and with the target height, whereas the difference between bone age and chronological age at the start and end of treatment was negatively correlated with FAH.

**Conclusion:**

FAH was significantly higher than the initial PAH in girls with CPP who were treated with GnRHa. Also, GnRHa treatment was still effective even after 8 years of age in girls with CPP.

## Introduction

Central precocious puberty (CPP) has been defined as the development of secondary sexual characteristics following the activation of the hypothalamic-pituitary-gonadal axis before the age of 8 years in girls and 9 years in boys [1]. Since 1981, Gonadotropin-releasing hormone agonist (GnRHa) has been the first treatment option for patients with CPP, and its suppressive effect on the hypothalamic-pituitary-gonadal axis is well established [2]. The objective of GnRHa treatment is to prevent early closure of the growth plate and preserve the genetic growth potential within the range of target height (TH); preventing physical differences in healthy peers who have normal puberty may lead to psychological stability [3].

Many studies have reported that the GnRHa treatment of patients with CPP increased final adult height (FAH) by from 4 to 8 cm compared with untreated patients [4]. Several factors such as age at initiation of treatment, initial height, target height, and initial bone age may influence the FAH following GnRHa treatment [5]. In particular, treatment at an age younger than 6 years resulted in effective growth outcome [6]. However, some studies reported that the GnRHa treatment of girls with CPP resulted in a similar effect even after 8 years of age [7]. CPP in girls may be difficult to diagnose at an early stage because parents do not know the exact time of the breast development. Also, previous studies reported that it took about 1.5 years from the time parents first noticed the pubertal symptoms to obtaining a diagnosis of CPP [8, 9]. Therefore, it remains debatable whether treatment is appropriate for many of the girls with CPP who visit the hospital after age 8.

Therefore, we evaluated the FAH in girls with idiopathic CPP and investigated whether the timing of treatment initiation affects the growth outcome.

## Methods

### Subjects and methods

#### Subjects

This study included girls with idiopathic CPP who were treated with leuprolide acetate for at least 1 year at the Pediatric Endocrine Unit of Ajou University Hospital from March 2003 to December 2014 (n = 243). Forty-three girls attained FAH during the observation period subsequent to treatment cessation. Among the patients lost to follow-up, we contacted girls who had reached late adolescence or who were expected to reach their FAH. We notified them of the present study and invited them to be reevaluated (n = 41). A total of 84 girls with idiopathic CPP who were treated with leuprolide acetate were finally included in the study. We defined FAH as a height velocity of less than 1 cm/year and bone age above 14.5 years [10]. The criteria for the diagnosis of CPP were (1) objective breast enlargement before 8 years of age, (2) advancement of bone age more than 1 year over chronological age, (3) pubertal luteinizing hormone (LH) response (cutoff ≥ 5 IU/L) to the GnRH stimulation test [3]. Excluded from the study were girls with brain tumor, congenital adrenal hyperplasia or hypothyroidism, and girls who had undergone cranial irradiation.

#### Study design

The GnRH stimulation test was performed to assess the pubertal status of all patients at initial evaluation. Basal serum samples were obtained prior to GnRH (100 μg Relefact; Sanofi-Aventis, Frankfurt am Main, Germany) injection, and post-stimulation samples were collected 30, 45, 60, and 90 min after injection to measure the LH and FSH levels. Leuprolide acetate was administered every 28 days at a dose of 3.75 mg in girls weighing more than 30 kg, 2.5 mg in girls weighing between 20 and 30 kg, and 1.87 mg in girls weighing less than 20 kg for at least 1 year [4]. Data on height, weight, BMI, pubertal status, PAH, and bone age were collected every 6 months from the clinical charts and electronic medical records. Pubertal status (Tanner stages of breast development) was assessed and documented by two pediatric endocrinologists. LH level was determined 30 min after leuprolide acetate injection every 6 months during the treatment to monitor GnRHa suppression. LH levels less than 3 IU/L were considered as therapeutic suppression [11]. Treatment was discontinued if one of the following criteria were met: the chronological age of 11 to 11.5 years or bone age of 12 to 12.5 years. Bone age was estimated by the same investigators using an X-ray of the left hand according to the method developed by Greulich and Pyle [12]. BMI (body mass index) was calculated (weight/height^2^), and the BMI and height standard deviation score (SDS) to adjust for age and sex were calculated using LMS methods from a reference population of Korean children [13]. The Bayley and Pinneau method was used to determine the predicted adult height (PAH) [14]. Target height was calculated by subtracting 6.5 cm from the mid-parental height.

The protocol was approved by the institutional review board of Ajou University Hospital (AJIRB-MED-OBS-16-372), and written informed consent was obtained from all the subjects or their parents before FAH evaluation.

#### Laboratory measurements

Serum LH and FSH values were measured by an immunoradiometric assay (BioSource, Nivelles, Belgium). The detection limits of the LH and FSH assays were 0.1 and 0.2 IU/L, the intra-assay coefficients of variation (CVs) were 1.4–3.9% and 1.1–2.0%, and the inter-assay CVs were 3.4–8.0% and 2.4–4.4%, respectively. E_2_ was measured by radioimmunoassay with a detection limit of 5 pg/mL, intra-assay CV of 4.0%–7.0%, and inter-assay CV of 4.2%–8.1% (Coat-A-Coung, Diagnostic Products, Los Angeles, CA, USA).

#### Statistical analysis

All analyses were conducted using SPSS ver. 23.0 software (IBM Corp., Armonk, NY, USA). Data were presented as means and standard deviations. Within-patient changes were performed using the paired *t* test. To determine the outcome according to age at onset of treatment, we divided all patients into two groups based on whether they had received treatment before or after 8 years. We used the independent *t* test to identify differences among patients according to age at treatment initiation. We performed multiple linear regressions with a stepwise variable selection to detect significant associations with FAH. We considered *P* < 0.05 to be statistically significant.

## Results

The mean chronological age and bone age at diagnosis were 8.2 ± 0.6 years and 10.3 ± 0.8 years, respectively. The stage of breast development of the majority of subjects at initial treatment was Tanner stage 2 (n = 47, 56%), with 31 (37%) children at Tanner stage 4, and 6 (7%) children at Tanner stage 4. The mean peak LH and FSH levels after the GnRH stimulation test were 13.3 ± 14.0 IU/L and 12.1 ± 4.1, respectively. The peak LH/FSH ratio was 1.14 ± 1.05. The basal and peak E_2_ levels were 10.3 ± 5.3 and 11.1 ± 5.4 pg/mL, respectively. The mean duration of GnRHa treatment was 2.98 ± 0.73 years (range: 1.5–4.8 years). All girls had menstruation after treatment cessation. Menarche occurred at 12.6 ± 0.6 years of age, which was 16.5 ± 6.1 months (range: 6–40 months) following GnRHa therapy.

The single LH levels determined 30 min after depot leuprolide acetate were suppressed (< 3 IU/L) in all girls during GnRHa treatment, indicating adequate hormone control. During treatment, the growth velocity was 5.2 ± 0.7 cm/year. Treatment was interrupted at 11.2 ± 0.5 years, and the girls grew 11.8 ± 3.8 cm after discontinuation of the treatment. The mean FAH was 160.1 ± 5.0 cm. The FAH was significantly greater (3.9 ± 4.6 cm) than the pretreatment PAH, but not significantly greater than PAH at the end of treatment or at target height (Table 1). In addition, BMI SDS was not significantly different between baseline (0.53 ± 0.92 SDS) and the end of treatment (0.57 ± 0.88 SDS). Furthermore, BMI SDS was significantly lower during FAH evaluation than at baseline or at treatment cessation.

**Table 1:** Clinical and auxological characteristics of 84 girls with central precocious puberty at baseline, the end of GnRHa treatment, and final adult height.

	Before treatment	At end of treatment	At adult height
Age (years)	8.2 ± 0.6	11.2 ± 0.5	14.1 ± 0.8
Height (cm)	132.7 ± 5.5	148.3 ± 6.1	160.1 ± 5.0*
Height SDS	1.12 ± 0.86	0.52 ± 0.79	-0.14 ± 1.02
Weight SDS	0.63 ± 0.57	0.69 ± 0.84	0.22 ± 1.07
BMI SDS	0.53 ± 0.92	0.57 ± 0.88	0.29 ± 1.06*
Bone age (year)	10.4 ± 0.8	11.8 ± 0.4	14.9 ± 0.2
BA-CA (year)	2.14 ± 0.75	0.57 ± 0.59	0.75 ± 0.77
PAH (cm)	156.1 ± 5.7	160.0 ± 5.9	
Target height (cm)	159.3 ± 3.7		

BA-CA, difference between bone age and chronological age; BMI, body mass index; PAH, predicted adult height; SDS, standard deviation score

* *P*<0.05 compared with pretreatment

To investigate whether growth outcomes were influenced by age at the initiation of treatment, we divided all patients into two groups, girls treated between 6 and 8 years (n = 23) and those treated after 8 years (n = 61); no significant differences were observed in FAH between the two groups. Further, the differences between FAH and the initial PAH or target height were not significantly different between the two groups (Table 2).

**Table 2:** Clinical and auxological characteristics of groups according to age at initiation of GnRHa therapy.

	Age at initiation of treatment		*P*
	6–8 yr (n = 23)	≥ 8 yr (n = 61)	
**Before treatment**			
Age (year)	7.4 ± 0.4 (6.2–7.9)	8.5 ± 0.2 (8.0–8.9)	<0.001
Height (cm)	129.4 ± 5.5	133.9 ± 4.9	<0.001
Height SDS	1.43 ± 1.00	1.01 ± 0.79	0.045
BA-CA (year)	2.50 ± 0.89	2.01 ± 0.64	0.022
PAH (cm)	156.0 ± 6.5	156.2 ± 5.5	0.880
Target height (cm)	159.6 ± 4.4	159.3 ± 3.5	0.694
**At end of treatment**			
Age (year)	11.1 ± 0.55	11.2 ± 0.57	0.291
Height (cm)	149.2 ± 7.0	147.9 ± 5.8	0.397
Height SDS	0.75 ± 0.92	0.44 ± 0.72	0.100
BA-CA (year)	0.69 ± 0.60	0.53 ± 0.58	0.253
PAH (cm)	161.1 ± 6.5	159.6 ± 5.7	0.326
Duration (year)	3.68 ± 0.66	2.72 ± 0.57	<0.001
**At final adult height**			
Age (year)	13.8 ± 0.64	14.2 ± 0.83	0.053
Bone age (year)	14.8 ± 0.20	14.9 ± 0.28	0.120
Height (cm)	160.6 ± 5.5	159.9 ± 4.9	0.617
ΔFAH-PAH at start (cm)	4.55 ± 5.79	3.71 ± 4.11	0.915
ΔFAH-TH (cm)	0.94 ± 4.72	0.8 ± 4.2	0.527

BA-CA, difference between bone age and chronological age; FAH, final adult height; PAH, predicted adult height; SDS, standard deviation score; TH, target height

Stepwise multiple regression analysis demonstrated that FAH SDS was significantly and positively correlated with height SDS at the end of treatment and with target height, whereas the difference between bone age and chronological age (BA-CA) at the start and end of treatment was negatively correlated with FAH (Table 3). Age at baseline, BMI SDS, duration of treatment, and PAH at start of treatment were not correlated with FAH.

**Table 3:** Factors associated with final adult height (SDS) in girls treated with GnRHa for precocious puberty (n = 84, r^2^ = 0.911, *P*<0.001).

Predictive factor	ß	Standard error	*P*
Height at the end of treatment (SDS)	1.257	0.055	<0.001
Target height	0.050	0.010	<0.001
BA-CA at start	-0.264	0.057	<0.001
BA-CA at the end of treatment	-0.939	0.071	<0.001

Stepwise multivariate regression analysis of the following independent variables: age at initiation of treatment, height SDS at start, BMI SDS, target height, BA-CA at start, height SDS at the end of treatment, duration of treatment, BA-CA at the end of treatment and PAH at start

BA-CA, difference between bone age and chronological age; BMI, body mass index PAH, predicted adult height; SDS, standard deviation score; TH, target height

## Discussion

In our study, the adult height of girls with idiopathic CPP who were treated with GnRHa was greater than their initial PAH before treatment by approximately 3.9 cm, and was close to their target height. Furthermore, we found no significant difference in height gain in groups according to the age at the onset of treatment. In addition, height SDS at the time of treatment cessation and advancement of bone age were significant predictors of FAH.

A number of researchers have investigated FAHs after long-term treatment of children diagnosed with CPP. The majority of these authors reported that height gain, which is the difference between the FAH and the initial predicted adult height, was between 2.0 cm and 9.8 cm, similar to the results obtained in our study [6, 7, 15–21]. Most authors analyzed the adult height compared with PAH based on patients’ bone age before treatment. Klein et al. [17] reported that FAH was 9.8 ± 9.0 cm greater than initial PAH before treatment in 80 girls with CPP. In another study, adult height showed a 4.8 ±5.8 cm increase compared with pretreatment PAH in 58 CPP girls [7]. In a recent study, the height gain in girls with CPP who were treated with GnRHa was significantly greater than that in untreated CPP girls by approximately 3 cm [22]. Lazar et al. [23] assessed the auxological outcomes of 142 women with CPP aged 27 to 50 years who either were treated with GnRHa or were untreated. The mean adult height of the GnRHa-treated women was significantly greater than that of the untreated women.

The effect of GnRHa treatment on height gain is well known. However, whether treatment is associated with the beneficial effects on height in girls who started treatment after 6 years of age or more is unknown. Most studies have reported that earlier age at initial GnRHa treatment was associated with greater adult height or with height gain [6, 18, 24]. Lazar et al. [6] divided the girls with CPP into three groups according to age at the start of treatment (before 6 years, between 6 and 8 years, and between 8 and 9 years) and reported that girls treated before 6 years achieved the greatest FAH. In another study, subjects who started treatment between the ages of 6 and 8 years showed significantly greater FAH than their initial PAH, although subjects who started treatment before 6 years of age were significantly taller than those who started after 6 years of age [17]. However, some studies showed no correlation between FAH and age or between height gain and age at initial treatment [7, 10, 15, 25]. Carel et al. [7] reported that 42 girls with pubertal onset between the ages of 6 and 8 showed a significant increase in FAH compared with initial PAH; age at onset of puberty or at onset of treatment was not correlated with height gain or adult height. Pasquino et al. [15] reported that height gain and FAH were not significantly different between girls treated before 7 and after 7. Michillo et al. [25] demonstrated improvement in FAH in girls treated after 8 years. In our study, we found no significant differences in height gain or FAH between the patients treated between 6 and 8 years and those treated after 8 years. These results may depend on the number of subjects, race, interpretation of bone age, and different treatment protocols such as treatment duration and cessation.

Various factors have been reported to affect height gain or adult height in girls with CPP. The factors reported to predict height gain or FAH were younger chronological age, the degree of bone age advancement, height SDS at start or end of treatment, and the duration of treatment [3, 4, 26]. In addition, factors associated with FAH were a greater PAH at start or end of treatment, target height, and growth velocity during treatment [27–29]. In our study, the degree of bone age advancement at treatment start and end and height SDS at treatment end were significantly correlated with FAH, similar to the results reported in other studies.

Our study has several limitations. The main limitation was the lack of untreated controls with CPP because of ethical issues or healthy age-matched controls. Also, we only evaluated height gain determined by the achieved adult height minus PAH based on bone age. Furthermore, we failed to include girls with CPP of before 6 years of age in this study.

In conclusion, our results confirm that GnRHa treatment improved height gain and FAH in girls with CPP. Also, girls with CPP may have visited the hospital after 8 years of age, although breast development began between 6 and 8 years of age; based on our results, these girls may still need to undergo effective treatment even between ages 8 and 9. Therefore, we suggest that treatment should be considered in girls diagnosed with CPP between ages 8 and 9. In addition, the monitoring of height and bone age during GnRHa treatment is important for predicting FAH.

## Supporting information

S1 File Main data set. (SAV)
